# List of Surgical Cases Treated in the Pennsylvania Hospital, and Discharged between the 1st and 23d of April, 1839

**Published:** 1839-04-20

**Authors:** J. Randolph

**Affiliations:** Attending Surgeon


### CLINICAL REPORTS.

*List of Surgical Cases treated in the Pennsylvania Hospital, and discharged between the* 1st *and* 23*d of April*, 1839. Dr. J. Randolph, Attending Surgeon.

[Reported by J. F. Meigs, M. D., Resident Surgeon.]

Admitted Feburary 19th, 1839, a case of fracture of both bones of the leg; treated by the fracture-box until the 21st of March, when pasteboard splints were applied. Discharged cured, April 1st, 1839, forty-one days after the accident.

Admitted February 6th, 1839, a case of compound fracture of the olecranon process, in which the joint was believed to be open, also a wound in the leg, situated between the tubercle of the tibia and the lower edge of the patella, produced by the explosion of a blast. The wounds being both small, and the man of middle age and good habit, it was determined to attempt to save the arm. It was placed on an angular splint, kept perfectly quiet, leeched, and cold applied. The leg was placed in a fracture-box and dressed with cerate. The man did well until the middle of March, when the arm was attacked with erysipelas, with an immense collection of pus, dissecting up the integuments of the arm; a collection of matter also took place in the knee joint. Porter, quinine, and full diet was given, but he sunk gradually, and died on the 5th of April, worn out by the discharge and irritation.

Admitted March 10th, a case of fracture of both bones of the fore-arm. In this case there was a strong tendency in the radius to approach the ulna, which, had it occurred, would of course have very much impeded the motions of the arm, by preventing the pronation and supination. It was combated successfully, however, by means of long narrow compresses, placed in the length of the fore-arm, and which, by forcing the muscles down between the two bones, obviated their tendency to approach. This case was discharged cured, on the 20th of April.

Admitted March 15th, a case of fracture of the olecranon, from a fall. Dressed with a straight splint on the front of the arm. The man left the hospital on the 1st of April, before the case was completed.

On the 1st of April a man was brought into the hospital, who, a few hours previously, had been run over the chest by a heavily laden cart; there was great difficulty of breathing, and a severe stitch in the right side. Over the middle of the sixth rib there was a slight emphysematous swelling, and at one time it was thought that crepitus could be felt, though not after the first day. A tight roller was applied; he was attacked next day with slight pleurisy, for which he was cupped; took a purge of calomel, with an opiate, and recovered so as to walk on the 8th. In this case the fracture of the rib was not well marked, neither did it incommode him after the first week, when the slight pleurisy was relieved. He left the house on the 10th of April.

A case of contusion of the shoulder, from a fall, was admitted, and dicharged cured in nine days, by means of rest and cold applications.

